# Supplementary material for: Genotypic and Phenotypic Characterization of Pathogenic Escherichia coli, Salmonella spp., and Campylobacter spp., in Free-Living Birds in Mainland Portugal
Source: Int J Environ Res Public Health. 2022 Dec 23;20(1):223. doi: 10.3390/ijerph20010223 (PMC9819048; doi:10.3390/ijerph20010223)
Supplement: Supplementary file 1 [file ijerph-20-00223-s001.zip › Supplementary Material Table S2_birds.pdf]

Isolate ID	Year	Animal	Pathotype	AMR phenotype	Serotype	Virulence genes	Resistance genes	Plasmids	MLST	Sample ID	ENA run Accession
E. coli											
Ec-PBG33	2021	*Columba livia*	STEC	SMX, TET, TMP	O45:H2	astA , cba , cif , cma, eae , espA , espB , espF , gad , hra , iss , nleA , nleB , nleC , ompT , stx2f , terC , traT	sul1 , tet(A), dfrA1	IncFIB, IncFII, IncI1-I	ST20	PT_EC0069	ERR9955189
EC-PBG48	2021	*Larus* spp.	EPEC	Susceptible	O125ac:H6	chuA , cif , eae, espA , espC , gad , ibeA , nleB , ompT , terC , tir , yfcV	Not found	Not found	ST583	PT_EC0070	ERR9955019
EC-EVOA41	2020	*Acrocephalus scirpaceus*	ExPEC	AMP, CHL, CIP, TET	O101:H9	astA , cea , gad , iss , terC , traT	blaTEM-1B , cmlA1 , qnrB19 , qnrB56 , qnrB67 , qnrB82 , tet(A)	Col, Col8282, IncFII, IncX1	ST10	PT_EC0061	ERR9954999
EC-EVOA45	2020	*Acrocephalus scirpaceus*	ExPEC	AMP, CHL, CIP, TET	O101:H9	astA , cea , gad , iss , terC , traT	blaTEM-1B , cmlA1 , qnrB19 , qnrB56 , qnrB67 , qnrB82 , tet(A)	Col, Col8282, IncFII, IncX1	ST10	PT_EC0062	ERR9955144
EC-EVOA54	2020	*Gallinula chloropus*	ExPEC	AMP, CHL, CIP, TET	O101:H9	astA , cea , gad , iss , terC , traT	blaTEM-1B , cmlA1 , qnrB19 , qnrB56 , qnrB67 , qnrB82 , tet(A)	Col, Col8282, IncFII, IncX1	ST10	PT_EC0063	ERR9954977
EC-EVOA55	2020	*Gallinula chloropus*	EAEC	Susceptible	O92:H33	aaiC , aap , aar , aggA , aggB , aggC , aggD , aggR , fyuA , gad , iha , irp2 , iss , iucC , iutA , mchB , mchC, mchF , ORF3 , ORF4 , pic , sat , terC	Not found	IncFIB	ST34	PT_EC0064	ERR9954980
EC-E60	2020	*Anas crecca*	STEC	Susceptible	O105:H7	vtx2f, eae, espA, espF, gad, iss, lpfA, nleA, nleB, nleC, ompT, terC, tir, traT	Not found	IncFIB	ST13581	PT_EC0065	ERR9955121
Ec-P2-I	2020	*Larus* spp.	ExPEC	AMP, CAZ, CIP, COX, FEP, NAL, SMX, TET, TMP	O55:H10	cvaC, etsC, fyuA, gad, hlyF, iroN, irp2, iss, iucC, iutA, lpfA, mchF, ompT, papC, sitA, terC, traT	blaTEM-1B, blaSHV-12, qnrB19, gyrA, sul2, tet(B), dfrA17	IncFIB, IncFIC, IncI1-I	ST162	PT_EC0054	ERR9955128
Ec-P4-I	2020	*Larus* spp.	ExPEC	AMP, CAZ, CIP, COX, FEP, NAL, SMX, TET, TMP	OND:H16	cma, cvaC, etsC, fyuA, gad, hlyF, iroN, irp2, iss, iucC, iutA, kpsE, lpfA, mchF, ompT, papC, sitA, terC, traT	blaTEM-1B, blaSHV-12, gyrA, sul2, tet(B), dfrA14	Col440II, IncFIB, IncFII, IncI1-I	ST453	PT_EC0055	ERR9955188
Ec-P6-I	2020	*Larus* spp.	ExPEC	AMC, AMP, AZM, CAZ, CIP, COX, FEP, NAL, SMX, TET, TMP	OND:H9	fyuA, gad, irp2, iucC, iutA, lpfA, senB, sitA, terC	blaCTX-M-15, blaTEM-1A, blaOXA-1, mph(A), aac(6')-Ib-cr , gyrA, sul1, sul2, tet(A), dfrA17	Col156, IncFIA, IncFIB, IncFII, IncI1-I, IncX4	ST410	PT_EC0056	ERR9955089
Ec-P16-I	2020	*Larus* spp.	ExPEC	AMP, CAZ, CIP, COX, FEP, NAL, SMX, TET	O78:H4	cia, cib, cvaC, etsC, fyuA, gad, hlyF, hra, iha, iroN, irp2, iss, iucC, iutA, lpfA, mchB, mchC, mchF, ompT, sitA, terC, tsh	blaCTX-M-1, gyrA, sul2, tet(A)	IncFIB, IncFIC, IncI1-I	ST23	PT_EC0057	ERR9955175
Ec-P17-I	2020	*Larus* spp.	EAEC	AMC, AMP, CAZ, COX, FOX	O111:H21	aaiC, aap, aar, afaD, agg3C, agg3D, agg5A, aggR, astA, espI, fyuA, gad, iha, iroN, irp2, iss, iucC, iutA, lpfA, ompT, ORF3, ORF4, pic, sat, sepA, terC, traT	ampC -promoter, blaTEM-1B	IncB/O/K/Z, IncFIB, IncFII	ST40	PT_EC0058	ERR9955174
Ec-P21-I	2020	*Larus* spp.	ExPEC	AMC, AMP, AZM, CAZ, CIP, COX, FEP, GMN, NAL, SMX, TET, TMP	O25:H4	chuA, cnf1, fyuA, gad, hra, iha, irp2, iss, iucC, iutA, kpsE, kpsMII_K5, mcbA, ompT, papA_F43, papC, sat, senB, sitA, terC, traT, yfcV	blaCTX-M-15, blaSHV-55, blaOXA-1, mph(A), aac(6')-Ib-cr, aac(3)-IIa, gyrA, qnrS1, sul1, tet(A), dfrA14, dfrA17	IncFIA, IncFII, IncN	ST131	PT_EC0059	ERR9955023
Ec-P22-I	2020	*Larus* spp.	ExPEC	AMC, AMP, AZM, CAZ, CIP, COX, FEP, GMN, NAL, SMX, TET, TMP	O25:H4	chuA, fyuA, gad, iha, irp2, iss, iucC, iutA, kpsE, kpsMII_K5, ompT, papA_F43, sat, sitA, terC, traT, yfcV	blaCTX-M-15, blaSHV-55, blaOXA-1, mph(A), aac(6')-Ib-cr, aac(3)-IIa, gyrA, qnrS1, sul1, tet(A), dfrA17	IncB/O/K/Z, IncFIA, IncFII, IncY	ST131	PT_EC0060	ERR9955093

Isolate ID	Year/Month	Animal	Serotype	AMR phenotype	MLST	Resistance genes	Plasmids	Sample ID	ENA run Accession
Salmonella enterica									
Se-PBG3	2020	*Circaetus gallicus*	IIIb 60:k:e,n,x,z15	Susceptible	ST3127	aac(6')-Iaa	Not found	PT_SE0147	ERR10115471
Se-PBG10	2020	*Columba livia*	Typhimurium	Susceptible	ST19	aac(6')-Iaa	IncFIB, IncFII, IncX1	PT_SE0148	ERR10115475
Se-PBG25	2021	*Columba livia*	Litchfield	Susceptible	ST214	aac(6')-Iaa	Not found	PT_SE0165	ERR10368807

Isolate ID	Year/Month	Animal	Species	AMR phenotype	MLST	Resistance genes	ENA run Accession
Campylobacter							
Cc-EVOA24	2020	*Acrocephalus scirpaceus*	*C. coli*	Susceptible	ST11400	Not found	ERR10372412
Cc-EVOA36	2020	*Fulica atra*	*C. coli*	Susceptible	ST11401	Not found	ERR10372474
Cj-EVOA54	2020	*Gallinula chloropus*	*C. jejuni*	AMP, CIP, TET	ST990	gyrA _T86I , tetO , blaOXA-466	ERR10372605
Cj-PBG16	2020	*Larus* spp.	*C. jejuni*	Susceptible	ST1268	Not found	ERR10372745
Cj-PBG18	2020	*Larus* spp.	*C. jejuni*	Susceptible	ST8572	Not found	ERR10372542
